# Correction to: Ethnic Density and Mental Health: Does it Matter Whether the Ethnic Density is Co-ethnic or Multi-ethnic and How Important is Change in Ethnic Density?

**DOI:** 10.1007/s40615-024-02139-1

**Published:** 2024-09-11

**Authors:** Jiyeong Seo, Stephen Jivraj

**Affiliations:** Institute of Epidemiology and Health Care, University College London, London, UK

**Correction to: Journal of Racial and Ethnic Health Disparities**

10.1007/s40615-024-02071-4

The article “Ethnic Density and Mental Health: Does it Matter Whether the Ethnic Density is Co-ethnic or Multi-ethnic and How Important is Change in Ethnic Density?”, written by Jiyeong Seo and Stephen Jivraj, was originally published electronically on the publisher’s internet portal on 23 July 2024 with Open Access. With the cancellation of Open Access the copyright of the article changed to © W. Montague Cobb-NMA Health Institute 2024 with all rights reserved.

